# The role of alcohol extract of cranberry in improving serum indices of experimental metaproterenol‐induced heart damage in rats

**DOI:** 10.1002/fsn3.3616

**Published:** 2023-08-09

**Authors:** Kimia Salmasi, Ali Hassanpour, Bahram Amouoghli Tabrizi, Sina Moghaddam

**Affiliations:** ^1^ Faculty of Veterinary Medicine, Tabriz Medical Science Branch Islamic Azad University Tabriz Iran; ^2^ Department of Clinical Sciences, Faculty of Veterinary Medicine, Tabriz Medical Science Branch Islamic Azad University Tabriz Iran; ^3^ Department of Internal Medicine, Faculty of Veterinary Medicine University of Tehran Tehran Iran

**Keywords:** cardiac function biomarkers, cardiotoxicity, cranberry, metaproterenol

## Abstract

Cranberry offers numerous cardiovascular benefits. According to several studies, this fruit promotes the oxidation of low‐density lipoprotein, enhances high‐density lipoprotein, reduces platelet coagulation, and improves vascular activity. Albino male rats were divided into five groups (*n* = 5 per group). The control group received intraperitoneal administration of normal saline. The second group was injected with metaproterenol (MET) 3 days a week for 4 weeks. The third, fourth, and fifth groups were given cranberry extract in doses of 75, 100, and 150, respectively, along with heart‐damaging drugs. Blood samples were collected and sent to the laboratory on the fourth weekend and 1 week after completing the injections in the fourth week (the sixth weekend) for analyzing serum factors such as cardiac creatine kinase MB, cardiac troponin I (cTnI), and aspartate aminotransferase (AST). The serum activity of the cardiac evaluation parameters in the fourth week demonstrated a highly significant correlation among the groups with respect to AST and cTnI (*p* < .001). Additionally, a significant relationship was observed between AST and cTnI within the target groups (*p* < .05). Ultimately, the findings indicated that the consumption of cranberry extract, due to its impact on heart function, could effectively modify serum indicators associated with heart damage. The utilized extract also exhibited efficacy, albeit with variable effects. Therefore, it is recommended to use cranberry extract synergistically with other chemical and herbal medications to achieve more sustained effects.

## Graphical abstract

Administration of cranberry extract effectively modifies serum indices associated with heart damage in rats. Cranberry extract effectively improves heart damage by reducing the release of these serum factors.

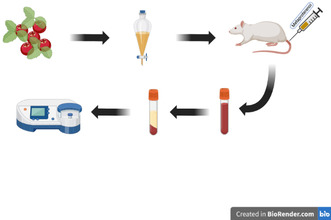

## 1 INTRODUCTION

Cardiovascular diseases, especially ischemic heart disease and heart attack, are considered the leading cause of global mortality, disability, and job loss. According to the Global Burden of Disease, cardiovascular patients have almost doubled from 1990 to 2019, and their mortality rate has increased from 12 million in 1990 to 18 million in 2019 (Roth et al., 2020). Today, one of the reasons for high mortality rates among noninfectious patients is injury and cardiovascular diseases, accounting for the death of millions of individuals annually in developing and developed countries (Pourmasoumi et al., 2020).

Cranberry belonging to the Ericaceae family is consumed in most countries and possesses antioxidant activities. Cranberry decreases the risk of cardiovascular diseases and has countless cardiovascular benefits (Diarra et al., 2020). According to previous studies, this fruit causes oxidation of low‐capacity lipoprotein and improves high‐capacity lipoprotein, reduces platelet coagulability, and improves vascular activity (El‐Belbasy et al., 2021). Myocardiotoxic drugs such as allylamine, cyclosporin A, doxorubicin (DOX), isoproterenol (ISO), and MET cause skeletal muscle damage, which can be detected by the AST and creatine kinase (CK) activities (Oda & Yokoi, 2021). In recent years, micro‐RNAs have been potential biomarker candidates for tissue damage. MiRNA‐208 is described in the heart, and miRs‐1 and miRs‐133a/b are developed in skeletal and heart muscles compared to other tissues. In current study compared the level of miRNA‐208, miRs‐1, and mirs‐133a/b with traditional tissue damage biomarkers; in cardiac (cTnI and FABP3) and skeletal muscle serum biomarkers (MYL3, sTnI, and AST) in rat administered several hearts and muscle toxication consist of ISO, MET, allylamine, and mitoxantrone. ISO and MET are catecholamines and nonselective β‐adrenergic receptors causing heart and skeletal muscle necrosis in long‐term usage (Calvano et al., 2016). As a result of damage to the myocardial, a large concentration of diagnostic myocardial infarction markers is released into the extracellular fluid (Ebenezar et al., 2003). These enzymes and macromolecules leaked from the damaged tissue are the best diagnostic indicators of tissue damage (Hearse, 1979). Due to the high prevalence of cardiovascular diseases in Iran, further studies are recommended to control and prevent such diseases (Sarrafzadegan & Mohammmadifard, 2019).

This study aimed to investigate the effect of cranberry extract on the serum levels of cTnI, creatine kinase MB (CK‐MB), and AST in rats with experimental heart damage with MET.

## 2 MATERIALS AND METHODS

### 2.1 Chemicals

Cranberry (Vaccinium macrocarpon) extract: This formulation was created by utilizing dried cranberry fruits and an alcoholic solvent. Metaproterenol sulfate (Alupent®, Iran) is purchased from the drugstore.

### 2.2 Experimental animals

Albino male rats weighing 120–150 g and 6 weeks old were obtained from the Faculty of Veterinary Medicine of the Islamic Azad University of Tabriz. The tested animals were kept at a standard temperature under humidity conditions in the research center of the Islamic Azad University of Tabriz. A suitable diet was provided to the mice.

### 2.3 Experimental design

Albino male rats were divided into five groups (*n* = 5 per group). In the control group, normal saline was administered intraperitoneally. The second group was injected with metaproterenol (MET) 3 days a week for 4 weeks. The third, fourth, and fifth groups received 75, 100, and 150 doses of cranberry extract and heart‐damaging drugs. Then the blood samples were taken and sent to the laboratory on the fourth weekend and a week after finishing the injections in the fourth week (the sixth weekend) to check the serum factors, including cardiac CK‐MB, cardiac troponin I (cTnI), and aspartate aminotransferase (AST) (Calvano et al., 2016; Galal et al., 2019; Hussien et al., 2015).

### 2.4 Serum collection for analysis

Twenty‐four hours after the last dose of specific treatment, half of the animals were anesthetized; the blood samples were obtained, and serum was separated by centrifugation for 10 min at room temperature. Two weeks later (in the sixth week), the process was repeated for the other half of the rats. Cranberry extract was not used from week 4 to week 6 to evaluate the stability of the cranberry's effect in rats.

### 2.5 Biochemical assays

Following the serum separation, the levels of cTnI, CK‐MB, and AST were evaluated using special commercial kits (Pars Azmoun, Iran) and an autoanalyzer device (WS‐ROCHE 912, Roche Hitachi, Japan).

### 2.6 Measurement of serum cTnI

The quantitative measurement of troponin is underpinned by the immune‐quantitative luminescence sandwich method. A monoclonal antibody covers the solid phase (magnetic particles), and a polyclonal antibody is used for the tracer. During the incubation of the troponin in the calibrator, the sample or control is attached to the solid phase of the monoclonal antibody. Subsequently, the conjugated antibody reacts with the troponin attached to the solid phase. This test must be performed in the LIAISON® analyzer. The analysis operation includes the following steps: 100 μL of a serum sample, calibrator or control, 200 μL of tracer conjugate, 20 μL of coated magnetic particles, 10‐min incubation and subsequent washing cycle, measurement within 3 s.

### 2.7 Measurement of serum cardiac CK‐MB

CK‐MB is a dimer enzyme consisting of two subunits, M (muscle) and B (brain), which combine to form the CK‐MM, CK‐MB, and CK‐BB isozymes. In this method, by using a specific antibody, the activity of the M subunit is inhibited, and CK‐MB corresponding to the activity of the remaining B subunit is measured by the CK‐NAC method. Since CK‐MB consists of two identical subunits, its activity is obtained by multiplying the obtained value by 2. The CK‐MB measurement is basically similar to the cTnI measurement test.

### 2.8 Measurement of serum AST

This reaction is underpinned by the optimized method proposed by ECCLS, which is the same as the IFCC method with no pyridoxal being used.

### 2.9 Data analysis

The collected data were analyzed with SPSS software version 24. The ANOVA test was used to compare the mean scores of the groups. In this study, *p* < .05 was set as the significance level.

## 3 RESULTS

Tables 1 and 2 present the serum activity of AST, cTnI, and CK‐MB 4 and 6 weeks after consuming cranberry alcoholic extract in the positive control and negative control groups, and the group receiving 75, 100, and 150 mg/kg doses. Duncan's postvariance test results also confirmed the variance analysis results.

**TABLE 1:** Comparison of mean serum parameters 4 weeks after consuming cranberry alcoholic extract in rats with heart damage using MET.

Serum parameters	Groups	Number	Mean ± standard error	*p*‐value
AST (U/L)	Positive control	5	95.00 ± 2.38	<.001
	Negative control	5	150.50 ± 12.60	
	Recipient dose 75 mg/kg	5	130.00 ± 2.55	
	Recipient dose 100 mg/kg	5	110.75 ± 1.10	
	Recipient dose 150 mg/kg	5	90.25 ± 2.89	
CK‐MB (U/L)	Positive control	5	406.00 ± 2.16	.202
	Negative control	5	707.75 ± 198.58	
	Recipient dose 75 mg/kg	5	567.75 ± 52.55	
	Recipient dose 100 mg/kg	5	456.50 ± 51.37	
	Recipient dose 150 mg/kg	5	436.00 ± 17.52	
cTnI (ng/mL)	Positive control	5	0.21 ± 0.006	<.001
	Negative control	5	0.31 ± 0.004	
	Recipient dose 75 mg/kg	5	0.30 ± 0.002	
	Recipient dose 100 mg/kg	5	0.27 ± 0.006	
	Recipient dose 150 mg/kg	5	0.25 ± 0.006	

Abbreviations: AST, aspartate aminotransferase; CK‐MB, creatine kinase MB; cTnI, cardiac troponin I; MET, metaproterenol.

**TABLE 2:** Comparison of mean serum parameters after 6 weeks in rats with heart damage using MET.

Serum parameters	Groups	Number	Mean ± standard error	*p*‐value
AST (U/L)	Positive control	5	90.25 ± 2.09	<.001
	Negative control	5	150.50 ± 12.60	
	Recipient dose 75 mg/kg	5	154.25 ± 17.83	
	Recipient dose 100 mg/kg	5	160.00 ± 5.87	
	Recipient dose 150 mg/kg	5	169.50 ± 3.37	
CK‐MB (U/L)	Positive control	5	396.25 ± 2.52	.301
	Negative control	5	707.75 ± 198.58	
	Recipient dose 75 mg/kg	5	575.75 ± 98.57	
	Recipient dose 100 mg/kg	5	524.00 ± 73.57	
	Recipient dose 150 mg/kg	5	442.00 ± 29.27	
cTnI (ng/mL)	Positive control	5	0.25 ± 0.004	.016
	Negative control	5	0.31 ± 0.004	
	Recipient dose 75 mg/kg	5	0.30 ± 0.004	
	Recipient dose 100 mg/kg	5	0.31 ± 0.008	
	Recipient dose 150 mg/kg	5	0.36 ± 0.04	

Abbreviations: AST, aspartate aminotransferase; CK‐MB, creatine kinase MB; cTnI, cardiac troponin I; MET, metaproterenol.

According to the findings, there was no significant relationship between the time of consumption of the cranberry alcoholic extract (4 or 6 weeks) and the serum variations of the measured parameters (Table 3).

**TABLE 3:** Correlation between measured serum parameters and time of receiving cranberry alcoholic extract in rats suffering from heart damage using MET.

Serum parameters	Correlation level (fourth and sixth week)
AST (U/L)	0.451
CK‐MB (U/L)	0.395
cTnI (ng/mL)	0.035

Abbreviations: AST, aspartate aminotransferase; CK‐MB, creatine kinase MB; cTnI, cardiac troponin I; MET, metaproterenol.

## 4 DISCUSSION

The present study evaluated the beneficial effect of cranberry extract on the cardiotoxicity caused by metaproterenol in laboratory mice.

Similar to some of their reported antitumor activities, the antioxidant properties of phenolics in cranberry fruit play a major role in the observed ability to reduce cardiovascular and age‐related diseases. Cranberry's role in preventing oxidative processes included a decrease in the oxidation of lipoproteins. Cranberry ranks high among fruits in antioxidant quality and quantity for their inherent flavonoid content, including proanthocyanidins, anthocyanins, flavonols, and phenolic acids (Galal et al., 2019).

Kalın et al. conducted a study in 2015 under the title Antioxidant activity and polyphenol content of cranberries (*Vaccinium macrocarpon*). Kalın et al.'s study helps to understand the antioxidant properties of blueberry extract and the presence of specific polyphenolic compounds. Our study provides insights into the effect of cranberry extract on cardiac function and its potential as a modifier of serum indices associated with cardiac injury. While both studies support the idea that cranberry extract offers health benefits, particularly in relation to cardiovascular health and antioxidant effects. Studies show that cranberry extract can be used synergistically with other drugs or antioxidants to increase effectiveness and create lasting effects. More research and exploration into the potential health benefits of cranberry extract are warranted (Kalin et al., 2015).

The diagnosis of heart damage and myocardial infarction is made by evaluating cardiac marker enzymes, including CK, CK‐MB, AST, alanine aminotransferase (ALT), lactate dehydrogenase (LDH), alkaline phosphatase (ALP), and cholesterol (Chrostek & Szmitkowski, 1989; Muhammad et al., 2011). These enzymes do not include contractile proteins and are not found in the bloodstream. In the case of myocardial necrosis, these are released into the blood (Acikel et al., 2005).

In previous studies, the induction of ISO, MET, and allylamine to rats caused heart damage (Calvano et al., 2016). In the present study, the induction of MET in rats increased parameters indicating cardiac damage. In some cases, increased cTnI represents ischemic damage induced by increased oxygen consumption, decreased blood pressure (perfusion), and decreased oxygen supply to the heart muscle (Ammann et al., 2001). The superiority of measuring cardiac troponins compared to other commonly used indicators has made them a gold standard for diagnosing myocardial infarction. Troponins are sensitive and specific indicators even for small amounts of myocardial necrosis (Coudrey, 1998). In the present study, cTnI levels significantly increased in the negative control group compared to the positive control group in the fourth and sixth weeks.

Compared to the control group, an excessive increase in CK and CKMB concentrations caused by DOX in serum indicates myocardial damage. The present findings are consistent with Afsar et al.'s reports, indicating that MET, including DOX, increases the serum activity of the mentioned parameters (the most basic biomarkers of myocardial cell damage; Afsar et al., 2017).

The normalization of CK, CK‐MB, and AST serum values in the tested groups receiving cranberry compared to those receiving heart‐damaging drugs suggested that cranberry extract could improve cardiac function. The findings of this study are in line with previous findings regarding the protective effect of plant extracts on cardiotoxicity caused by DOX (Eman et al., 2011).

Many studies have reported that rutin has a protective effect on the heart in case of myocardial infarction caused by ISO induction (Annapurna et al., 2009). In the present study, cranberry extract had favorable protective effects on the heart in rats damaged by metaproterenol. This healing effect on the heart can be justified by reducing serum parameters in the groups receiving the extract.

Troponin 1 is one of the cardiac regulatory proteins and improves the contractile function of the heart (Sharma et al., 2004). In the present study, cTnI was present only in the myocardium; therefore, it was used as a marker of cardiac damage. When the heart cell dies, this protein is released from the heart into the bloodstream. Hence, the level of this serum parameter was higher in animals receiving MET than in the group not receiving this drug. The level of cTnI, however, was low in the groups receiving both heart‐damaging drugs and cranberry extract, suggesting that the cranberry extract can improve cardiac function.

Cardiac marker enzymes include AST, CK‐MB, and cTnI and act as markers for diagnosing myocardial damage (Lim et al., 2013). Our study showed a significant decrease in the levels of these serum parameters in the groups receiving cranberry extract with a dose of 150 mg/kg compared to the negative control group. Cranberry extract has an effect even in a low dose; as the dose increases, its effect on improving cardiac function also increases. Accordingly, cranberry plays a comprehensive role in preventing cardiac damage and reducing the parameters of cardiac damage.

This study also showed the oxidative damage caused by increased free radicals in the heart tissues after MET administration. Damage to the heart myocardium leads to the release of serum parameters of cardiac indicators such as AST, CK‐MB, and cTnI in the blood, leading to the diagnosis of cardiac damage (Nimbal & Koti, 2017).

Cardiac cell damage significantly decreased in rats receiving different doses of cranberry extract reduced compared to those receiving MET. As a result of heart damage, the level of cTnI, one of the most reliable and common biomarkers, increased. However, the effects and longevity of cranberry extract were not permanent. After stopping the administration of this extract, the values of the cTnI parameter were higher in the group receiving 150 doses of this extract in the sixth week than in the negative control group. This implies that the stability of cranberry extract is short, and it should be consumed for a more prolonged period (Henri et al., 2016; O'brien et al., 2006).

In Kharadi et al.'s study, the administration of Allium cepa aqueous extract at a dose of 400 mg/kg resulted in the recovery of increased parameters (troponin I, CK‐MB, and AST; Kharadi et al., 2016). In our study, the administration of cranberry extract in 75, 100, and 150 doses led to the recovery of the aforementioned parameters, especially in the fourth week. The recovery of troponin I, CK‐MB, and AST by this extract was significant in the present study.

CK enzymes, especially CK‐MB, convert ATP into ADP and transfer energy to cardiac myosin filaments. The sensitivity of CK‐MB measurement is 95% in many studies, and it is a highly specific marker in confirming cardiac damage (Hettling & van Beek, 2011; Muralidharan et al., 2008). A remarkable increase in this enzyme was observed in the studied rats with heart damage. While the level of this enzyme decreased in rats receiving different doses of cranberry extract. This implies that cranberry extract is a strong cardiac protector inhibiting cardiac necrosis caused by MET.

## 5 CONCLUSION

It can be concluded that consuming cranberry extract with its effect on heart function can effectively correct serum indicators related to heart damage. The tested extract effectively improved heart damage by reducing the release of these serum factors. As the effect of this extract was not stable, after stopping the administration of this extract for 2 weeks, serum factors unfortunately reincreased in the sixth week, suggesting that the used extract was also effective; however, its effects were not stable. Accordingly, it is recommended to be used synergistically with other chemical and herbal medicines to achieve more prolonged effects.

## AUTHOR CONTRIBUTIONS

**Kimia Salmasi:** Conceptualization (equal); data curation (equal); formal analysis (equal); investigation (equal); methodology (equal); project administration (equal); resources (equal); validation (equal). **Ali Hassanpour:** Conceptualization (equal); data curation (equal); formal analysis (equal); funding acquisition (equal); investigation (equal); methodology (equal); project administration (equal); resources (equal); software (equal); supervision (equal); validation (equal); visualization (equal); writing – original draft (equal); writing – review and editing (equal). **Bahram Amouoghli Tabrizi:** Conceptualization (equal); data curation (equal); formal analysis (equal); investigation (equal); methodology (equal). **Sina Moghaddam:** Conceptualization (equal); data curation (equal); formal analysis (equal); investigation (equal); methodology (equal); project administration (equal); resources (equal); software (equal); supervision (equal); validation (equal); visualization (equal); writing – original draft (equal); writing – review and editing (equal).

## FUNDING INFORMATION

Not applicable.

## CONFLICT OF INTEREST STATEMENT

The authors report no conflict of interest.

## ETHICS STATEMENT

The protocol of this study was developed according to the ethical principles approved by the international committees for the protection of the rights of laboratory animals (Code: IR.IAU.TABRIZ.REC.1400.120).

## Data Availability

All the data supporting the findings of this study are included in the article.
